# The omicron variant of SARS‐CoV‐2: Understanding the known and living with unknowns

**DOI:** 10.1002/ctm2.685

**Published:** 2021-12-15

**Authors:** Nicholas E. Ingraham, David H. Ingbar

**Affiliations:** ^1^ Pulmonary Allergy Critical Care, and Sleep Medicine Division Department of Medicine University of Minnesota Twin Cities Minneapolis Minnesota USA

**Keywords:** COVID‐19, Omicron, pandemic, variant, virus

## Abstract

The recently discovered Omicron variant of SARS‐CoV‐2 has rapidly burst into the public and scientific eye, being detected in more than 26 countries around the world. Given its more than 50 mutations, there is widespread concern about its public health impact, leading the World Health Organization to designate it a variant of concern. This Commentary provides a summary of current knowledge and unknowns about this viral variant as of December 2, 2021 and summarizes the key questions that need to be rapidly answered.

## 1 INTRODUCTION

The SARS‐CoV‐2 virus has infected over 260 000 000 people globally with over 5 000 000 people dying from the coronavirus disease‐2019 (COVID‐19) over the past 2 years since it was first discovered.^1^

A herculean effort has been put forth to combat COVID‐19 through scientific research, public health and patient care.^2^ Unlike many diseases, where preventative measures and treatment change over decades, the list of new COVID‐19 treatments and vaccines grows on the scale of days and weeks. The need for swift action comes from the contagious nature of this new virus with little prior immunity. An important contribution is the virus’ predilection for mutations which may undue the prior successes over the past 2 years. One of the most concerning mutations (based on the number of mutations, where they occurred and early spread) was recently discovered, the Omicron variant.^3^

### 1.1 SARS‐CoV‐2 structure, pathophysiology and variants

This Coronavirus is an enveloped virus with a positive‐sense single‐stranded RNA genome and a nucleocapsid of helical symmetry.^4^ It has three major proteins. The envelope (E) protein is a minor structural protein that forms pentameric ion channels. The membrane (M) protein is the main structural protein and the spike (S) protein consists of head (S1) and stem (S2) subunits in a 3:2 ratio. The N terminal portion of the S1 heterotrimer usually binds to cell surface carbohydrates while the C terminal domain recognizes protein receptors on cell surfaces, including angiotensin‐converting enzyme 2 (ACE2), aminopeptidase N, or dipeptidyl peptidase 4.

The Coronavirus life cycle starts with the S protein binding to cell surface receptors followed by host cell protease cleavage of the receptor‐attached S protein, activating it, and resulting in fusion of viral and host cell membranes. The single‐stranded viral RNA hijacks host synthetic machinery to make virus‐specific RNA polymerase, new viral genomes, and other viral proteins. Viral M protein integrates into the endoplasmic reticulum (ER) and promotes the assembly of new viral particles as an ER complex that are released as new virions by exocytosis.

Within less than 2 years, many mutated variants of the original SARS‐CoV2, identified in Wuhan, China and designated 614G, have been recognized. Variants are assessed from epidemiologic, clinical, pathophysiologic, and therapeutic viewpoints. Factors using an epidemiologic lens include transmission, the magnitude of viral load, duration of shedding and rate of viral load depletion after its zenith. Clinical variables include time course, symptoms, risk factors, clinical duration, severity and long‐term sequelae. Variants may have altered diagnostic properties in rapid antigen or polymerase chain reaction (PCR) tests and altered interactions with cell surface receptors and/or the immune system. Finally, variants may differ in response to particular therapies (mitigation strategies).^5^

Recognition and knowledge about variants are rapidly expanding and individual variants typically include multiple different mutations in inconsistent combinations. Using the SARS‐CoV‐2 interagency group variant classification, at present there are no Variants of high consequence; two Variants of concern (Delta and Omicron), no Variants of Interest and 10 additional Variants being monitored.^6^ The B1.1.7 variant initially was recognized in the United Kingdom in September 2020 with its first detection in the United States in late December 2020. The mutations are in the receptor‐binding domain of the spike protein (N501Y and several other mutations).^7^ It spreads more rapidly with possibly some increased severity and mortality than 614 G, but no decrease in protection by vaccines. The B.1.351 variant was first detected in South Africa in October 2020 and the United States in late January 2021. It includes a variety of spike protein mutations (K417N; E484K or N501Y), but has no major difference in transmission or severity. There has been some concern about possibly less antibody neutralization of this variant and lower preventive efficacy of the Astra Zeneca vaccine. The P.1 variant initially was identified on routine screening of travelers from Brazil to Japan and is related to the B.1.1.28 variant lineage. It includes at least 17 unique mutations with some occurring in the spike protein receptor‐binding domain. It may have somewhat lesser recognition by antibodies, and there is concern about possible increased transmission and reinfection. The Delta variant (B.1.617.2) has become the predominant variant in much of the world during the second half of 2021. The Delta variant has at least a 40%–60% increase in transmissibility,^8^ resulting in higher viral loads^9^ and increased hospitalizations in unvaccinated people.^10^ The AY.1 and AY.2 lineages are less benefited by treatment with monoclonal antibodies.^11^

This article will summarize what is currently known about the recently discovered Omicron variant, shed light on why there is major concern, and identify the key questions about Omicron that need to be answered in the coming months to appropriately position the response to the marked public fears about Omicron as a new Variant of Concern.

### 1.2 Mutations

#### 1.2.1 Known

Omicron has at least 50 mutations identified, with 32 of those mutations involving the spike protein when compared to the reference strain.^12^, ^13^, ^14^ For comparison, the delta variant has nine spike protein mutations along with 13 others.^14^, ^15^ Of these 50 mutations, 26 are unique, further building upon the 10 unique mutations seen in Delta and six from Beta. As described previously, the environment may be prime for viral adaptation.^16^ When both immune pressure (from vaccinations) and viral abundance are present at moderate levels, the rate of variant evolution peaks along its theoretical bell curve.^16^

Many of the most concerning mutations occur in the virus's receptor‐binding domain. These mutations may affect the spike protein's affinity for ACE 2 (ACE2). The binding ability to ACE2 remains a critical aspect of the virus's pathogenicity.^17^ The specific concern is when mutations enhance their binding ability to these cell surface receptor proteins.^18^ The Omicron variant has two mutations that may increase the spike protein's affinity to ACE2, contributing to the current concern.^19^ Other specific Omicron mutations have a theoretical risk of increasing transmissibility (three mutations at the cleavage site),^20^ while another set of mutations may result in evasion of the immune system.^21^

#### 1.2.2 Unknown

An important caveat to the description of mutations listed above is that many of the 'concerns' with each mutation remain theoretical. The interplay between all the mutations in tandem and the resultant clinical manifestations are key to assessing what comes next. While the risks that this mutation causes a higher R^o^ (basic reproduction number) or more severe disease are based on scientific knowledge, the real‐world impacts must be appropriately evaluated. Understanding how the Omicron virus will spread, infect and evade will only truly be answered in retrospect from accumulating clinical data. The current testing and surveillance will remain vital to informing the degree of public health measures that are needed while we wait. Furthermore, the public health priorities to respond to Omicron will largely be determined by the transmissibility and severity of this variant.

### 1.3 Where is it?

#### 1.3.1 Known

As of December 1st, 2021, at least 27 countries in six continents have identified the Omicron variant, including the United States.^22^ South Africa and Botswana were the first countries to identify the variant in mid‐November, in part based on samples from November 9, 2021. This discovery stemmed from their phenomenal efforts in South Africa to sequence the SARS‐CoV‐2 virus at a higher rate than most countries. The global community is indebted to them for being extremely proactive and vigilant. In retrospect, Omicron was also found in the Netherlands in mid‐November 2021. It likely also was present in other countries at that time. This presumed early identification has given the global community a chance to shift their focus to this variant at a very early stage. Most PCR tests detect the Omicron variant with a different ‘signature’ from the Delta variant. This may provide the fortuitous opportunity to assess the spread and frequency of Omicron based on PCR testing, even before full sequencing is completed.

#### 1.3.2 Unknown

Importantly, patient zero may never be identified. Furthermore, we can say with certainty that we do not know where the variant first emerged. This point is critical given the polarizing environment that we live in; focusing on scientific successes and clear messaging must remain a priority going forward.^2^ Sequencing takes time (multiple days to weeks) and is resource‐intensive. There is significant variation across different countries in their capability and rates of sequencing a significant fraction of SARS‐CoV‐2 isolates in an ongoing fashion, and antigen test‐diagnosed cases are not available for sequencing. With the very recent recognition of Omicron and the variable frequency of sequencing, the prevalence of Omicron likely will be underrepresented for a significant period. At this point, Omicron is likely widespread and present in most countries. The degree to which it spreads will depend on many of the unknown clinical characteristics, mainly transmissibility and its ability to compete with the current dominant strains (primarily Delta) across the globe.

### 1.4 Infectivity and transmissibility

#### 1.4.1 Known

Very little is known about either infectivity or transmissibility at this time. Anecdotal situations may provide some insight. For example, the several airline flights from South Africa to the Netherlands with subsequently detected COVID infections of passengers may provide epidemiologic experiments of nature. There is a major concern that Omicron may have high infectivity and transmissibility due to its sudden appearance in South Africa with rapid recognition in other countries. The rapid upsurge in COVID‐19 cases in South Africa coincident with the discovery of Omicron suggests high transmissibility – although the prevalence of Omicron in driving this upsurge is not yet known. Very preliminary modeling of South Africa data (with the assumption that Omicron is driving the surge) from two separate groups derived similar R_t_ (secondary cases from an index case) estimates of 0.8‐2.5.^23^, ^24^ Importantly, R_t_ is a combination of R_0_ and the ability of a variant to escape the immune system.^25^, ^26^ Given that Omicron contains mutations linked to both transmissibility and immune escape, it may be a combination of both aspects that drive Omicron's presumed dominance over other strains. At this early stage, there has been a patchwork public health and governmental response, with some countries shutting their borders to all non‐citizen entry and some others excluding flights from Africa.

#### 1.4.2 Unknown

Aside from the presence of mutations with theoretical implications of increased transmissibility and increasing COVID‐19 cases from an ecological standpoint, no definitive statements about infectivity or transmission are possible at present. The current uptick of cases in areas with known Omicron may, in fact, turn out to be the canary in the coal mine, but until there is an increase in genetic sequencing across the globe, and the large degree of patient‐ and local‐level confounding factors (e.g., vaccination rates, booster rates, public health measures, population density, age distribution) are accounted for, reliable conclusions cannot be reached.^27^ Determination of the true rate of transmissibility will likely take weeks and will require each region to help determine the prevalence of the variant and whether current mitigation strategies are being implemented efficaciously (i.e., mask type, early monoclonal antibody therapy).

### 1.5 Clinical illness severity and lethality

#### 1.5.1 Known

Most of the early reported cases of Omicron infections are anecdotal statements from South African doctors about young adult patients with mild clinical symptoms, not requiring hospitalization or critical care.^28^ Some of these patients had reinfections, raising the possibility of lesser protection from either prior COVID infection. However, given the lack of reliable surveillance of patients for Omicron, this well may change. The first United States case reported December 1, 2021, from California also had a mild illness in a previously vaccinated individual. Thus far, Omicron‐infected patients requiring intensive care with intubation have not yet been recognized and reported at this very early time since discovery.

#### 1.5.2 Unknown

The disease severity and any unique clinical profile of Omicron will be gradually determined as anecdotal reports from around the world are combined and data accumulates for subgroups, such as high risk, unvaccinated, previously infected and boosted patients.

### 1.6 Vaccine efficacy

#### 1.6.1 Known

It is estimated that it will take at least 2–3 weeks to appropriately assess the neutralizing capacity of our current interventions (vaccines and convalescent plasma) through in vitro testing and longer to determine in vivo protection.^29^ Some of the mutations are in spike protein regions that are immune‐dominant and thought to be important in antibody‐mediated host defense. Other mutations are in domains that are targeted by T cell‐mediated host defense. Scientists from South Africa have shared their belief that prior infection with COVID‐19 provides relatively little immune protection against subsequent infection with the Omicron variant.^30^ Fortunately most of the anecdotally reported Omicron cases thus far have had relatively mild symptoms and clinical manifestations. Vaccine manufacturers rapidly launched major efforts to create mRNA vaccines modified to be effective against the Omicron variant, but it is too early to be able to assess the immune response and/or protective efficacy.

#### 1.6.2 Unknown

Almost all elements of vaccine efficacy in the prevention or mitigation of Omicron infection are currently unknown. Even the CEOs of the two largest vaccine manufactures have differing thoughts on whether the vaccines remain as efficacious against Omicron as it has with the preceding strains.^31^ At this point, whether the vaccine efficacy remains strong or decreases, the most critical and efficacious tool in our armamentarium remains vaccinating the global population. Reported Omicron cases are in the hundreds while >99% of current COVID‐19 cases are from SARS‐CoV‐2 strains for which the vaccines were developed or work extremely well against. Reducing the prevalence of new SARS‐CoV‐2 infections, in general, should reduce the rate of emergence of further new post‐Omicron variants, that have equal or even greater potential adverse impact.

## 2 CONCLUSIONS

The information gained about the Omicron variant and actions taken over the next few weeks and months will be critical to ensuring success in combating the pandemic. The Omicron variant may become a dark period that includes a global surge or it may be a false alarm that quickly fades from memory. Either way, our actions now must be the same to ensure success in both scenarios. (1) Vaccinate the globe: The Omicron variant highlights the need to vaccinate the world which requires looking beyond our individual nations. (2) Clear and transparent communication: This must occur frequently between the scientific community and the public and in an equitable manner.^32^ Now, more than ever, is there a need to strike the balance of preparing for the worst and hoping for the best. (3) Collaboration: The scientific community has already broken many silos over the past 2 years, these collaborations must continue to thrive as new information emerges each day. Key questions about the Omicron variant that need answers are, 'What are the transmissibility and infectivity of this variant?', 'How effective is each of the vaccines in preventing or mitigating Omicron infection?', 'What are the severity, lethality and long‐term sequelae of Omicron infection?', 'How efficacious are currently used therapies in Omicron treatment (monoclonal antibody infusion, for example)?' and 'How much protection against Omicron infection and serious illness, including death, is conferred by prior COVID‐19 infection?'

## CONFLICT OF INTEREST

All authors have substantially contributed to conducting the underlying research and drafting this manuscript. To the best of our knowledge, none of the authors have a conflict of interest, financial, or otherwise.
